# Assessing the Gun Violence Archive and Public Police Data as Comprehensive Sources for Gun Violence

**DOI:** 10.1001/jamanetworkopen.2024.58054

**Published:** 2025-01-31

**Authors:** Michael G. Anchan, Jennifer Harris, Sophia Natale-Short, Megan R. Georges, Elizabeth C. Pino

**Affiliations:** 1 Boston University Chobanian & Avedisian School of Medicine, Boston, Massachusetts; 2 Boston Violence Intervention Advocacy Program, Department of Emergency Medicine, Boston Medical Center, Boston, Massachusetts; 3 Department of Emergency Medicine, Boston Medical Center, Boston, Massachusetts; 4 Department of Emergency Medicine, Boston Medical Center and Boston University Chobanian & Avedisian School of Medicine, Boston, Massachusetts

## Abstract

This cross-sectional study evaluates how many fatal and nonfatal firearm injuries in the Boston area are captured in the Gun Violence Archive and public police data and what factors are associated with being included in these datasets.

## Introduction

Gun violence has been recognized as a public health crisis in the United States.^1^ Nevertheless, no single, comprehensive database exists to track fatal and nonfatal firearm injuries.^2^ This study evaluates the comprehensiveness of the Gun Violence Archive (GVA) and public police data in capturing incidents of community and interpersonal gun violence using emergency department data as a reference standard.

## Methods

This cross-sectional study used data from a cohort of patients presenting to the Boston Medical Center emergency department (ED) for a non–self-inflicted firearm injury between 2019 and 2023 (eFigure in Supplement 1). ED patient data were matched to publicly available police department data (eTable in Supplement 1) and to the GVA based on several identifying factors. The Boston University institutional review board waived authorization for research and the requirement for informed consent because it was deemed impracticable considering the sample size and difficulty locating patients. We followed STROBE guidelines for cross-sectional research. Logistic regression models were used to evaluate the association between patient, injury, and incident characteristics and the primary outcomes of inclusion in the GVA and police databases (eMethods in Supplement 1).

## Results

Of the 766 patients (median [IQR] age, 28 [21-34] years; 698 [91.1%] male patients) presenting with firearm injuries (Table 1), most were transported to the hospital by ambulance (79.1%), and their gunshot wounds required hospital admission (59.0%) but were ultimately nonfatal (87.5%). Most patients were shot in single-injury incidents (75.5%) with no fatalities (86.7%) that took place within Boston (85.9%). Overall, 563 patients (73.5%) were captured by the GVA, and 685 patients (89.4%) were captured in public police data; 512 patients (66.8%) were included in both databases, and 30 patients (3.9%) were excluded by both databases.

**Table 1.** Characteristics of Individuals With Firearm Injuries Treated at Boston Medical Center (BMC) Who Were Included or Missing From the GVA and Police Databases, 2019-2023

Characteristic	Patients, No. (%)				
	Total (N = 766)	GVA		Police department	
		Included (n = 563 [73.5%])	Missing (n = 203 [26.5%])	Included (n = 685 [89.4%])	Missing (n = 81 [10.6%])
Age, median (IQR), y	28 (21-34)	28 (22-34)	27 (21-33)	28 (22-34)	25 (21-32)
Age group					
<20 y	123 (16.1)	86 (15.3)	37 (18.2)	114 (16.6)	9 (11.1)
≥20 y	643 (83.9)	477 (84.7)	166 (81.8)	571 (83.4)	72 (88.9)
Gender					
Women	68 (8.9)	51 (9.1)	17 (8.4)	61 (8.9)	7 (8.6)
Men	698 (91.1)	512 (91.0)	186 (91.6)	624 (91.1)	74 (91.4)
Race and ethnicity					
Black	526 (68.7)	390 (69.3)	136 (67.0)	483 (70.5)	43 (53.1)
Hispanic	162 (21.2)	111 (19.7)	51 (25.1)	145 (21.2)	17 (21.0)
White	32 (4.2)	22 (3.9)	10 (4.9)	21 (3.1)	11 (13.6)
Other^a^	18 (2.4)	16 (2.8)	2 (1.0)	17 (2.5)	1 (1.2)
Unknown	28 (3.7)	24 (4.3)	4 (2.0)	19 (2.8)	9 (11.1)
Injury characteristics					
Transport					
Walk-in	160 (20.9)	86 (15.3)	74 (36.5)	141 (20.6)	19 (23.5)
Ambulance	606 (79.1)	477 (84.7)	129 (63.6)	544 (79.4)	62 (76.5)
Admitted to hospital					
No	314 (41.0)	223 (39.6)	91 (44.8)	281 (41.0)	33 (40.7)
Yes	452 (59.0)	340 (60.4)	112 (55.2)	404 (59.0)	48 (59.3)
Fatal					
No	670 (87.5)	468 (83.1)	202 (99.5)	595 (86.9)	75 (92.6)
Yes	96 (12.5)	95 (16.9)	1 (0.5)	90 (13.1)	6 (7.4)
Incident characteristics					
Multi-injury					
No	578 (75.5)	390 (69.3)	188 (92.6)	509 (74.3)	69 (85.2)
Yes	188 (24.5)	173 (30.7)	15 (7.4)	176 (25.7)	12 (14.8)
Total injured in incident, median (IQR) [range]	1 (1-1) [1-8]	1 (1-2) [1-8]	1 (1-1) [1-4]	1 (1-2) [1-8]	1 (1-1) [1-3]
Any fatalities in incident					
No	664 (86.7)	464 (82.4)	200 (98.5)	589 (86.0)	75 (92.6)
Yes	102 (13.3)	99 (17.6)	3 (1.5)	96 (14.0)	6 (7.4)
Occurring in Boston					
No	108 (14.1)	77 (13.7)	31 (15.3)	46 (6.7)	62 (76.5)
Yes	658 (85.9)	486 (86.3)	172 (84.7)	639 (93.3)	19 (23.5)
Year					
2019	141 (18.4)	112 (20.0)	29 (14.3)	127 (18.5)	14 (17.3)
2020	216 (28.2)	158 (28.1)	58 (28.6)	204 (29.8)	12 (14.8)
2021	176 (23.0)	110 (19.5)	66 (32.5)	148 (21.6)	28 (34.6)
2022	135 (17.6)	106 (18.8)	29 (14.3)	116 (16.9)	19 (23.5)
2023	98 (12.8)	77 (13.7)	21 (10.3)	90 (13.1)	8 (9.9)

Abbreviation: GVA, Gun Violence Archive.

^a^ Other race and ethnicity includes American Indian or Alaskan Native, Asian, Native Hawaiian or Pacific Islander, and all other races.

Firearm injuries were at higher odds of being described in the GVA if patients were transported to the ED by ambulance as opposed to walking in (odds ratio [OR], 2.72; 95% CI, 1.83-4.04; *P* < .001), if their injuries were fatal (OR, 28.67; 95% CI, 3.94-208.58; *P* = .001), and by increasing numbers of individuals injured in a single incident (OR per each additional person, 5.19; 95% CI, 2.84-9.50; *P* < .001) (Table 2). Firearm injuries were at higher odds of being described in public police databases if patients were transported by ambulance (OR, 2.68; 95% CI, 1.26-5.72; *P* = .01) and if the incident occurred within Boston compared with surrounding municipalities (OR, 44.30; 95% CI, 24.48-80.17; *P* < .001). Patient age, gender, and race and ethnicity were not associated with inclusion in either the GVA or police data.

**Table 2.** Factors Associated With Patients Treated for Firearm Injuries at Boston Medical Center Being Included in the GVA and Public Police Databases, 2019-2023

Characteristic	GVA				Police department			
	Univariate		Multivariable^a^		Univariate		Multivariable^a^	
	OR (95% CI)	*P* value	OR (95% CI)	*P* value	OR (95% CI)	*P* value	OR (95% CI)	*P* value
Year	1.00 (0.89-1.14)	.96	NA	NA	0.90 (0.75-1.08)	.25	NA	NA
Age group								
<20 y	0.81 (0.53-1.24)	.33	NA	NA	1.60 (0.78-3.29)	.20	NA	NA
≥20 y	1 [Reference]		NA		1 [Reference]		NA	
Gender								
Women	1 [Reference]	.77	NA	NA	1 [Reference]	.94	NA	NA
Men	0.92 (0.52-1.63)		NA		0.97 (0.43-2.19)		NA	
Race/ethnicity								
Black	1.30 (0.60-2.82)	.16	NA	NA	5.88 (2.66-13.01)	<.001	NA	NA
Hispanic	0.99 (0.44-2.24)		NA		4.47 (1.84-10.83)		NA	
White	1 [Reference]		NA		1 [Reference]		NA	
Other^b^	3.64 (0.70-18.92)		NA		8.90 (1.04-76.04)		NA	
Unknown	2.73 (0.75-9.97)		NA		1.11 (0.38-3.25)		NA	
Transport								
Walk-in	1 [Reference]	<.001	1 [Reference]	<.001	1 [Reference]	.55	1 [Reference]	.01
Ambulance	3.18 (2.21-4.59)		2.72 (1.83-4.04)		1.18 (0.68-2.04)		2.68 (1.26- 5.72)	
Admitted to hospital								
No	1 [Reference]	.20	NA	NA	1 [Reference]	.96	NA	NA
Yes	1.24 (0.90-1.71)		NA		0.99 (0.62-1.58)		NA	
Fatal								
No	1 [Reference]	<.001	1 [Reference]	.001	1 [Reference]	.15	NA	NA
Yes	41.00 (5.68-296.14)		28.67 (3.94-208.58)		1.89 (0.80-4.47)		NA	
Multi-injury incident								
No	1 [Reference]	<.001	NA	NA	1 [Reference]	.03	NA	NA
Yes	5.56 (3.19-9.69)		NA		1.99 (1.05-3.76)		NA	
No. injured in incident	3.76 (2.32-6.11)	<.001	5.19 (2.84-9.50)	<.001	1.71 (1.07-2.73)	.02	NA	NA
Any fatalities in incident								
No	1 [Reference]	<.001	NA	NA	1 [Reference]	.10	NA	NA
Yes	14.22 (4.46-45.40)		NA		2.04 (0.86-4.81)		NA	
Occurring in Boston								
No	1 [Reference]	.62	NA	NA	1 [Reference]	<.001	1 [Reference]	<.001
Yes	1.12 (0.71-1.76)		NA		44.30 (24.48-80.17)		44.30 (24.48-80.17)	

Abbreviations: GVA, Gun Violence Archive; OR, odds ratio; NA, not applicable.

^a^ Logistic regression models were used for estimating ORs, 95% CIs, and *P* values. Multivariable model for inclusion in the Gun Violence Archive was adjusted for method of transport to hospital, fatality of injury, and number of individuals injured by firearms in an incident (assessed as a continuous variable). Multivariable model for inclusion in public police data was adjusted for method of transport to hospital and whether the shooting incident occurred within Boston compared with surrounding municipalities.

^b^ Other race and ethnicity includes American Indian or Alaskan Native, Asian, Native Hawaiian or Pacific Islander, and all other races.

## Discussion

In this analysis, patients were less likely to be captured in the GVA if they self-presented to the ED, if their injuries were nonfatal, or if they were the only person injured in a shooting incident. Reliance on media sources for compiling GVA data omits more than a quarter of shooting injuries presenting to the ED, limiting its utility as an epidemiological data source.^3,4^

Police department databases captured a higher percentage of ED-presenting patients with gunshot wounds, with those self-presenting to the ED or injured outside of Boston less likely to be included. Boston Police Department (BPD) provides nearly comprehensive data within the city, capturing 97% of patients injured within Boston. The remaining 19 patients (3%) with firearm injuries occurring in Boston who were omitted included those injured due to legal intervention and those injured in locations outside of BPD jurisdiction, eg, shootings on city buses assigned to transit police and shootings on state park land assigned to state police. These exclusions underscore the limitations of an otherwise robust resource for firearm injury data in a major metropolitan area. Incomplete data from smaller police jurisdictions limits their use for state-wide analysis. This study was limited by only including patients treated at a single medical center and excluding firearm homicides occurring outside the hospital.

As a leading cause of death in the United States^5^ and a critical driver of health disparities,^6^ gun violence is a public health issue that requires accurate investigation. These findings highlight the necessity of establishing state-sponsored, comprehensive gun violence data infrastructure through linkage of multiple sources.

## Notes

Supplement 1.
eMethods.
**eFigure.** Flowchart of Analysis **eTable.** Summary of Police Department Data Sources
eReferences.

Supplement 2.
Data Sharing Statement
