# Supplementary material for: Assessing the Gun Violence Archive and Public Police Data as Comprehensive Sources for Gun Violence
Source: JAMA Netw Open. 2025 Jan 31;8(1):e2458054. doi: 10.1001/jamanetworkopen.2024.58054 (PMC11786224; doi:10.1001/jamanetworkopen.2024.58054)
Supplement: Supplement 1. — eMethods. eFigure. Flowchart of Analysis eTable. Summary of Police Department Data Sources eReferences. [file jamanetwopen-e2458054-s001.pdf]

Supplemental Online Content

Anchan MG, Harris J, Natale-Short S, Georges MR, Pino EC. Assessing the Gun Violence Archive and public police data as comprehensive sources for gun violence. *JAMA Netw Open.* 2025;8(2):e2458054. doi:10.1001/jamanetworkopen.2024.58054

eMethods.

eFigure. Flowchart of Analysis

eTable. Summary of Police Department Data Sources

eReferences.

This supplemental material has been provided by the authors to give readers additional information about their work.

© 2025 Anchan MG et al. *JAMA Network Open*.

eMethods.

Study Design and Participants

Boston Medical Center (BMC), the region's largest safety-net hospital, is an urban level I trauma center that treats approximately 70% of patients with gunshot wounds in the city of Boston.[1] Patients were identified from the BMC Violence Intervention Advocacy Program (VIAP) clinical database, as previously described.[2,3] Shooting incident locations were acquired from police, EMS, and patient self-report to the VIAP trauma response supervisor at the time of emergency department (ED) presentation.

ED patient data was matched to publicly available police department data and to the GVA based on several identifying factors: incident date, address, police district, time of day, total number of individuals injured or killed; patient name (if listed), age, gender, race, and ethnicity. For every ED-presenting gunshot wound patient in the VIAP database, author MGA attempted to match to an incident in the public police databases and the Gun Violence Archive (GVA). Author SNS separately confirmed all matches. For all incidents found not to have a match (e.g., an ED gunshot wound patient found to have no matching incident in the GVA), author ECP performed a final check for accuracy. A match in the GVA or police databases to the VIAP dataset was confirmed if the location of the shooting was identical, if the time of the shooting approximately matched, if the total number of individuals injured in the incident matched the hospital dataset, or if the gender, age, race/ethnicity, and fatality of the individual matched. If incomplete information was available on ED-presenting patients that could be matched to datasets, partially linked information was sufficient to affirm a match (e.g., if no incident address was available in the VIAP dataset, a match could be affirmed based on linked gender, number injured individuals, and Boston police district for a shooting on a specified date). This methodology could have potentially led to an underestimation of the shooting incidents missing from the GVA and police datasets.

Measures

Age was assessed as a continuous variable and dichotomized into categories of pediatric (less than 20 years old) and adult (20 years and older) patients. All patients were grouped with their affirmed gender for all analyses, including one transgender woman. We abstracted the self-identified race and ethnicity from the patient medical record. Race and ethnicity were queried from separate survey questions, such that we could categorize our population into five mutually exclusive racial/ethnic groups: non-Hispanic White, non-Hispanic Black, Hispanic (any race), other race (including Asian, American Indian or Alaskan Native, Native Hawaiian or Pacific Islander, and all other races), and those missing race/ethnicity information ("unknown"). Race and ethnicity were assessed due to the documented disparities in firearm violence[4] and in capturing incidents of firearm violence in the GVA[5].

Injury characteristics were categorized by method of transport to hospital (walk-in vs. ambulance), hospital admission, and fatality of injury. Incident characteristics were categorized by the total number of individuals injured by firearms in an incident, whether there were any fatalities in the incident, and the city where the shooting took place.

Data Sources

The GVA is one of the largest public databases for tracking gun violence in the US, utilizing media, police, government, and local sources to compile lists of shootings.[6] Automated queries on the internet and manual research are used to collect the incident locations of incidents in which a patient sustained a gunshot wound with the goal of providing a public research repository accurately depicting the impact of gun violence. For the purposes of this study, the database search tool function was utilized to identify all shooting incidents in Massachusetts between 2019 and 2023 in which an individual was fatally or nonfatally shot.

Publicly available police shooting databases from Boston and surrounding localities are described in eTable 1. For the city of Boston in which 85.9% of VIAP shooting incidents took place, we utilized information from the Boston Police Department (BPD) shootings dashboard[7] linked by incident number to the BPD crime incident reports[8]. The shootings dashboard contains information on shooting incidents where a victim was struck by a bullet, either fatally or non-fatally, that occurred in Boston and fall under BPD jurisdiction. Self-inflicted gunshot wounds or shootings BPD determined to be justifiable (i.e., legal intervention) are excluded. This database included information on shooting time and date, police district, fatality, multi-victim incidents, and gender, race, and ethnicity of the individual shot. Crime incident reports document the initial details surrounding an incident to which BPD officers respond, including information on offense type, date and time, and incident geolocation and street name. Outside of Boston, police departments from surrounding cities and towns offered varying degrees of information in publicly available police logs or logs available upon request. Many police departments had no public data readily accessible.

Statistical Analysis

Summaries of patient demographics and injury and incident characteristics were conducted over all patients and by inclusion in the GVA and police databases. Categorical variables were presented with frequencies and percentages and continuous variables were summarized as medians and interquartile ranges (IQR).

Univariate and multivariable logistic regression models were used to evaluate the association between the primary outcomes of inclusion in the GVA and police databases and patient, injury, and incident characteristics. Crude univariate odds ratios (ORs) with associated 95% confidence intervals (95% CIs) were derived for each exposure variable under study. We selected covariates for the multivariable model a priori based on previously published reports of factors associated with inclusion in public shooting databases[5,9,10] and availability of data. The full multivariable logistic regression model included all covariates listed in Table 1. The final multivariable logistic regression model for inclusion in the GVA included significant ($p < 0.05$) covariates from the full model: mode of transport to hospital, fatality of injury, and total number of individuals injured by firearms in the incident. The final multivariable logistic regression model for inclusion in public police databases included significant covariates from the full model: mode of transport to hospital and incident occurring within Boston. No violations of collinearity were observed among variables included in the final multivariable models. We assessed model fit using the Hosmer-Lemeshow Goodness-of-Fit Test. All analyses were conducted using Stata 18 (StataCorp). Statistical tests used 2-sided $P < .05$ as the threshold for significance.

© 2025 Anchan MG et al. *JAMA Network Open*.

eFigure 1. Flowchart of Analysis

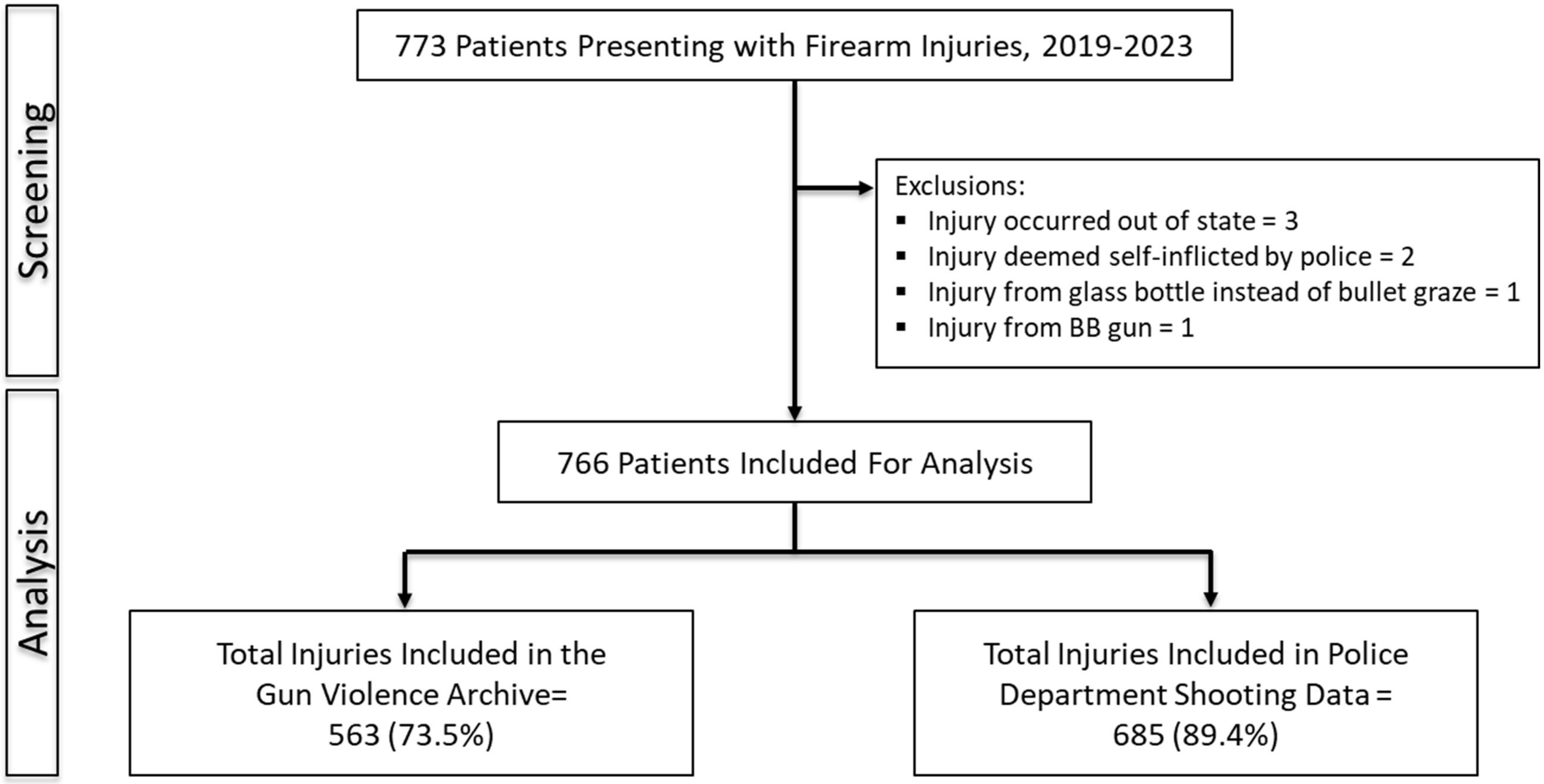

© 2025 Anchan MG et al. *JAMA Network Open*.

eTable. Summary of Police Department Data Sources

City	Shootings, n (%)[a]	Database Description	Variables Included
**Boston**	658 (85.9)	The Boston Police Department (BPD) Shootings Dashboard contains information on shooting incidents where a victim was struck by a bullet, either fatally or non-fatally, that occurred in the City of Boston and falls under Boston Police Department jurisdiction. The dashboard does not contain records for self-inflicted gunshot wounds or shootings determined to be justifiable (i.e. legal intervention shootings).<br><br>BPD Crime Incident Reports document the initial details surrounding an incident to which BPD officers respond and indicate if a shooting took place. BPD Incident numbers link incidents in the BPD Shootings Dashboard to incidents in the Crime Incident Reports.	Incident number, shooting date, BPD district, fatality of injury, victim gender, victim race, victim ethnicity, number of victims in incident.<br><br>Incident number, shooting date, BPD district, offense code, offense description, shooting time, shooting location – street, shooting location – geolocation.
**Abington**	1 (0.13)	The Abington Police Department Blotter documents police responses from December 20, 2021 to present in the town of Abington, MA. It provides call logs, but no further information on victim demographics.	Incident number, incident date, incident time, incident address, type of response, neighborhood.
**Avon**	1 (0.13)	The Avon Police Public Call Log documents calls that the police department responded to in the town of Avon, MA. It provides call logs, but no further information on victim demographics.	Call number, call taker, incident date, incident address, incident time, responding officer(s), EMS unit, fire unit, suspect name, suspect address, suspect age, suspect charges, incident number if police actions occurred, and call reason.
**Belmont**	1 (0.13)	The Belmont Police Blotter provides information regarding incident number, date, location, and type. Specific calls if not listed can be requested via pubic records request.	Incident number, incident date, incident address, incident time, incident type.
**Beverly**	1 (0.13)	The Beverly Police Logs provide information regarding daily calls. Logs are published for 30 days, after which they are consolidated into a yearly police log. After 30 days, call information may be obtained via written request.	Incident number, incident date, incident address, incident time, incident type, responding unit(s), call description.
**Braintree**	3 (0.39)	The Braintree Police Department does not publish a public log of calls or a call database. It does publish an arrest log. A call log was requested by the authors.	
**Brockton**	45 (5.87)	The Brockton Police Department Call Log contains information regarding 911 calls received by dispatchers and the associated police response. Logs do not specify whether the incident is a shooting unless the call is considered a gunshots call. Logs also do not provide any information on the nature of the call or victim status.	Call number, call taker, incident date, incident address, incident time, responding officer(s), suspect name, suspect address, suspect age, suspect charges, incident number if police actions occurred, and call category.
**Cambridge**	2 (0.26)	The Cambridge Police Department (CPD) publishes daily call logs which contain information regarding 911 calls fielded by the department, as well as press releases for more high acuity incidents, such as shootings. In addition, CPD releases an annual report, which highlights the number of fatal and non-fatal firearm incident, as well as victim and offender information for murders.	Incident number, shooting date, shooting time, shooting location, brief description of incident, criminal complaint and penal code, suspect name, victim name if fatal.

© 2025 Anchan MG et al. *JAMA Network Open*.

City	Shootings, n (%)[a]	Database Description	Variables Included
**Chelsea**	3 (0.39)	The Chelsea Police Department does not publish a public log of calls or a call database. It does publish an arrest log, a use of force database, and UCR Crime Statistics by month. None of these databases, however, specify whether a shooting has occurred, only if a non-fatal or fatal violent incident occurred.	
**Fall River**	2 (0.26)	The Fall River Police Department does not publish a public call log.	
**Falmouth**	1 (0.13)	The Falmouth Police Department maintains public police logs, arrest logs, and yearly crime reports.	Incident number, incident date, incident address, incident time, incident type.
**Framingham**	2 (0.26)	The Framingham Police Department Public Log provides information regarding incident number, date, location, and type. Specific call logs, if not listed, can be requested via pubic records request.	Date reported, incident time, call number, call type, incident address, and responding unit(s).
**Haverhill**	2 (0.26)	The Haverhill Police Department does not publish a public call log.	
**Lawrence**	2 (0.26)	The Lawrence Police Department publishes a police log which includes information on incidents including incident number, date, time, location, and type. Logs exist for each calendar day.	Incident number, incident date, incident type, incident location.
**Lowell**	1 (0.13)	The Lowell Police Department does not publish a public call log.	
**Lynn**	1 (0.13)	The Lynn Police Department publishes daily call logs. These logs contain information on police responses, but no information on the victim demographics, victim status, or mechanism of injury unless the call is a gunshots call.	Incident date, incident time, incident number, incident type, incident location.
**Malden**	1 (0.13)	The Malden Police Department provides weekly call logs.	Incident number, incident date, incident time, incident type, incident location, disposition, responding officer.
**Methuen**	1 (0.13)	The Methuen Police Department does not publish a public call log.	
**Milton**	1 (0.13)	The Milton Police Department publishes thorough police logs, which include incident dispatcher remarks.	Incident number, incident date, incident time, incident street, incident type, action taken, incident dispatcher remarks.
**Quincy**	8 (1.04)	The Quincy Police Department does not publish a public call log.	
**Randolph**	16 (2.09)	The Randolph Police Department publishes daily call logs. The authors requested and received additional logs for specific dates.	Incident number, incident date, incident time, incident street, caller reports, incident type, responding unit(s).
**Rockland**	1 (0.13)	The Rockland Police Department publishes monthly call logs.	Incident number, incident date, incident time, incident street, incident type, actions taken.
**Springfield**	1 (0.13)	The Springfield Police Department does not publish a public call log. The department publishes arrest logs.	
**Stoughton**	4 (0.52)	The Stoughton Police Department does not publish public call logs.	
**Taunton**	3 (0.39)	The Taunton Police Department provides call logs which cover part of the calendar years.	Incident number, incident date, incident time, incident street, incident type, actions taken.
**Weymouth**	1 (0.13)	The Weymouth Police Department maintains public call logs for part of the calendar year.	Call number, call time, call reason, call taker, incident location, and responding officers.

© 2025 Anchan MG et al. *JAMA Network Open*.

City	Shootings, n (%)[a]	Database Description	Variables Included
**Worcester**	2 (0.26)	The Worcester Police Department does not publish public call logs.	
**Yarmouth**	1 (0.13)	The Yarmouth Police Department does not publish public call logs.	

[a] Values are frequencies and percentages of total shootings in study population.

© 2025 Anchan MG et al. *JAMA Network Open*.

eReferences

1. Boston Medical Center. Injury Prevention Center Annual Report 2014-2015. https://www.bumc.bu.edu/emergencymedicine/files/2016/06/16114_IPC_AR_2016_web-FINAL.pdf. Published 2016. Accessed November 13, 2020.
2. Pino EC, Fontin F, Dugan E. Violence Intervention Advocacy Program and Community Interventions. In: Lee LK, Fleegler EW, eds. *Pediatric Firearm Injuries and Fatalities : The Clinician's Guide to Policies and Approaches to Firearm Harm Prevention.* Cham: Springer International Publishing; 2021:157-177.
3. Pino EC, Fontin F, James TL, Dugan E. Boston Violence Intervention Advocacy Program: Challenges and Opportunities for Client Engagement and Goal Achievement. *Acad Emerg Med.* 2021;28(3):281-291.
4. Kegler SR, Simon TR, Zwald ML, et al. Vital signs: changes in firearm homicide and suicide rates—United States, 2019–2020. *Morb Mortal Weekly Rep.* 2022;71(19):656.
5. Gobaud AN, Mehranbod CA, Kaufman E, et al. Assessing the gun violence archive as an epidemiologic data source for community firearm violence in 4 US cities. *JAMA network open.* 2023;6(6):e2316545-e2316545.
6. Gun Violence Archive. General Methodology. https://www.gunviolencearchive.org/methodology. Accessed May 7, 2024.
7. Boston Police Department. Shootings Dashboard. https://data.boston.gov/dataset/shootings. Accessed January 8, 2024.
8. Boston Police Department. Crime Incident Reports. https://data.boston.gov/dataset/crime-incident-reports-august-2015-to-date-source-new-system. Accessed January 8, 2024.
9. Kaufman EJ, Passman JE, Jacoby SF, et al. Making the news: victim characteristics associated with media reporting on firearm injury. *Prev Med.* 2020;141:106275.
10. Kaufman E, Holena DN, Yang WP, et al. Firearm assault in Philadelphia, 2005–2014: a comparison of police and trauma registry data. *Trauma surgery & acute care open.* 2019;4(1):e000316.

© 2025 Anchan MG et al. *JAMA Network Open.*
