# Supplementary material for: Assessing the Gun Violence Archive and Public Police Data as Comprehensive Sources for Gun Violence
Source: JAMA Netw Open. 2025 Jan 31;8(1):e2458054. doi: 10.1001/jamanetworkopen.2024.58054 (PMC11786224; doi:10.1001/jamanetworkopen.2024.58054)
Supplement: Supplement 2. — Data Sharing Statement [file jamanetwopen-e2458054-s002.pdf]

Data Sharing Statement

Anchan. Assessing the Gun Violence Archive and Public Police Data as Comprehensive Sources for Gun Violence. *JAMA Netw Open*. Published February 03, 2025. doi:10.1001/jamanetworkopen.2024.58054

Data
Data available: No

Additional Information
Explanation for why data not available: All data from the Gun Violence Archive and local police databases are already publicly available. Data from hospital records cannot be made publicly available due to HIPAA protections.
